# Genotypes and Mouse Virulence of *Toxoplasma gondii* Isolates from Animals and Humans in China

**DOI:** 10.1371/journal.pone.0053483

**Published:** 2013-01-07

**Authors:** Lin Wang, He Chen, Daohua Liu, Xingxing Huo, Jiangmei Gao, Xiaorong Song, Xiucai Xu, Kaiquan Huang, Wenqi Liu, Yong Wang, Fangli Lu, Zhao-Rong Lun, Qingli Luo, Xuelong Wang, Jilong Shen

**Affiliations:** 1 Department of Parasitology, Provincial Laboratory of Microbiology & Parasitology and the Key Laboratory of Zoonoses Anhui, Anhui Medical University, Hefei, Anhui, China; 2 Clinical Laboratory, the First Affiliated Hospital of Anhui Medical University, Hefei, Anhui, China; 3 Clinical Laboratory, the First Affiliated Hospital of Anhui University of Traditional Chinese Medicine, Hefei, Anhui, China; 4 Provincial Institute of Parasitic Diseases Control Anhui, Hefei, Anhui, China; 5 State Key Laboratory of Biocontrol, School of Life Sciences, the Key laboratory of Tropical Diseases Control, the Ministry of Education, and the Department of Parasitology, Sun Yat-Sen University, Guangzhou, Guangdong, China; 6 Department of Parasitology, Tongji Medical College, Huazhong University of Science and Technology, Wuhan, Hubei, China; 7 Department of Pathogen Biology, Nanjing Medical University, Nanjing, Jiangsu, China; 8 Department of Parasitology, and the Key Laboratory of Tropical Disease Control, Ministry of Education, Zhongshan School of Medicine, Sun Yat-sen University, Guangzhou, Guangdong, China; INSERM U1094, University of Limoges School of Medicine, France

## Abstract

**Background:**

Recent population structure studies of *T. gondii* revealed that a few major clonal lineages predominated in different geographical regions. *T. gondii* in South America is genetically and biologically divergent, whereas this parasite is remarkably clonal in North America and Europe with a few major lineages including Types I, II and III. Information on genotypes and mouse virulence of *T. gondii* isolates from China is scarce and insufficient to investigate its population structure, evolution, and transmission.

**Methodology/Principal Findings:**

Genotyping of 23 *T. gondii* isolates from different hosts using 10 markers for PCR-restriction fragment length polymorphism analyses (SAG1, SAG2, SAG3, BTUB, GRA6, c22-8, c29-2, L358, PK1 and Apico) revealed five genotypes; among them three genotypes were atypical and two were archetypal. Fifteen strains belong to the Chinese 1 lineage, which has been previously reported as a widespread lineage from swine, cats, and humans in China. Two human isolates fall into the type I and II lineages and the remaining isolates belong to two new atypical genotypes (ToxoDB#204 and #205) which has never been reported in China. Our results show that these genotypes of *T. gondii* isolates are intermediately or highly virulent in mice except for the strain TgCtwh6, which maintained parasitemia in mice for 35 days post infection although it possesses the uniform genotype of Chinese 1. Additionally, phylogenetic network analyses of all isolates of genotype Chinese 1 are identical, and there is no variation based on the sequence data generated for four introns (EF1, HP2, UPRT1 and UPRT7) and two dense granule proteins (GRA6 and GRA7).

**Conclusion/Significance:**

A limited genetic diversity was found and genotype Chinese 1 (ToxoDB#9) is dominantly circulating in mainland China. The results will provide a useful profile for deep insight to the population structure, epidemiology and biological characteristics of *T. gondii* in China.

## Introduction

*Toxoplasma gondii* is an obligate intracellular parasite of worldwide distribution which infects almost all warm-blooded animals, including humans [1]. Felids are important in the transmission of *T. gondii* to humans and animals because they are the only hosts that excrete the environmentally resistant oocysts in the faeces [2]. It is estimated that nearly one-third of the human population in the world have infection with toxoplasmosis [3]. Humans may acquire *T. gondii* infection via (i) oral uptake of food and water contaminated with oocysts shed in the faeces from infected cats; (ii) consumption of raw or undercooked meat with tissues cysts; (iii) transplacental transmission of tachyzoites from mother to the foetus. Primary infection in immunocompetent patients is mostly asymptomatic but in immunocompromised individuals it may cause life-threatening encephalitis due to the rupture of pre-existent cysts that reactivate latent infections [4], [5], [6]. *T. gondii* infection during pregnancy can lead to devastating disease for the foetus and newborn infant, particularly for women with inadequate prenatal care [7], [8], [9].

The distribution of *T. gondii* genotypes varies in geographic regions [10]. In North America and Europe, *T. gondii* has three main clonal lineages that are designated types I, II, and III, comprising the vast majority of isolates. Type I is highly virulent to out-bred mice and type II and III strains are significantly less virulent [11]. Moreover, the fourth clonal lineage, referred to as type 12, has recently been described and is the most common type in wildlife in North America [12], [13]. Archetypal isolates predominated in chickens in Africa that, as in North America and Europe, have high prevalence of types II and III [14], as well as the Africa 1 and Africa 3 are among the major types in Africa [12], [15]. In contrast, the isolates from humans and animals from tropical regions of South America are genetically diverse, and different from the ones found in North America and Europe [16], and severe toxoplasmosis in immunocompetent human patients is frequently associated with atypical genotypes in South America [17], [18]. All the data reported suggest that *T. gondii* propagates largely clonally in Europe, Africa and North America, but shows greater evidence of sexual recombination in South America [19]. Speculation about the global evolution of *T. gondii* has led to a worldwide effort to study genetic diversity within this fascinating organism [10].

China is located in the east of the Asia-Europe continent and occupies nearly fifty degrees of latitude between the north and the south territory. Animal biodiversity is rich, with a temperate and humid climate. There are fifty-six ethnic groups living in China and some of them have their own unique habits of eating raw meat that potentially increases the risk of *T. gondii* infection. This may have particular consequences if infection occurs in immunocompromised individuals and pregnant women [20], [21]. The seropositive rate in human populations of this parasite was found to be 7.9% based on serological investigations in China, [22], and four genotypes have been identified from animals [23], [24], [25]. There are no data, however, on genotypes and virulence of *T. gondii* isolates from humans in China. To achieve this it is necessary to have more strains of *T. gondii* from animals and humans to understand the biological and genetic characteristics of *T. gondii*. The objectives of the present study were to investigate the genotypes and their associated virulence of *T. gondii* in animals and humans in China.

## Results

### Serological Prevalence and Patients Data

All patients analyzed in the study had clinical and/or laboratory diagnoses of toxoplasmosis. They had, at least, positive PCR, or CAg, or IgG and/or IgM antibodies against *T. gondii*. Serological detection was performed on sera of 118 human patients and 11 cases (9.3%, 11/118) were positive, and four of them were found to have positive CAg and IgM antibodies, positive *T. gondii* PCR and viable tachyzoites (Table 1).

**Table 1:** The medical data from patients with toxoplasmosis.

Patient No	Isolate	Location	Age(yr)/sex of patient^a^	Immune status/Medical history^b^	Sample	Time since onset of symptoms
1	TgHuAh1	WuHu, Anhui	66/M	IC/HCC	blood	4 months
2	TgHuAh2	WuHu, Anhui	45/M	IC/HCC	blood	2 months
3	TgHuZSE	Guangzhou, Guangdong	25/F	IC/AIDS	blood	1 months
4	TgHuZS2	Guangzhou, Guangdong	60/M	IC/AIDS	blood	1 months

a F, female; M, male;

b IC, immunocompromised; HCC, hepato-cellular carcinoma;

In the present study, the genotyping of *T. gondii* was carried out with the isolates from the immunocompromised patients admitted and treated at different public hospitals in Anhui and Guangdong Provinces of China. The data from four patients (3 males and 1 female) are presented in Table 1. These patients presented characteristics of ocular lesions suggestive of Toxoplasmic retinochoroiditis. Among them were two cases of AIDS and two cases of hepato-cellular carcinoma (HCC) with long term chemotherapy of oxaliplatin (OXA) combined with 5- Fluorouracil (5- Fu) for six weeks.

### Genetic Characterization of Chinese *T. gondii* Isolates

Twenty-three strains that killed mice for parasites passage, together with 14 isolates taken from the earlier collection [26], were subject to analyses of genotypes and virulence. Genotyping results of the 23 *T. gondii* isolates at 10 genetic loci revealed five genotypes and these were compared to those listed in ToxoDB; three of the five genotypes were atypical, and two were archetypal (Table 2). The atypical genotypes were designated as Chinese 1 (ToxoDB#9), ToxoDB#204 and ToxoDB#205, respectively. Fifteen of the 23 samples (65.22%) from humans and animals in different regions of China (with distances between each other greater than 400 km) belong to Chinese 1 lineage. The genotype Chinese 1 showed type II patterns at SAG2, GRA6, L358, PK1, c22-8, but c29-2, SAG3, BTUB loci displayed a type III pattern and type I at the Apico locus. Genotype Chinese 1 had been previously identified from humans and animals in south and central China [27], suggesting that this genotype is the major lineage and is obviously widespread throughout mainland China.

**Table 2:** Multilocus PCR-RFLP genotyping of *T. gondii* from animals and humans in China.

Isolates ID	Host	SAG1	SAG2	SAG3	BTUB	GRA6	c22-8	c29-2	L358	PK1	Apico	ToxoDB PCR-RFLP Genotype	Comments
GT1	Goat	I	I	I	I	I	I	I	I	I	I	**#10 (Type I)**	Reference
PTG	Sheep	Π/Ш	Π	Π	Π	Π	Π	Π	Π	Π	Π	**#1 (Type II)**	Reference
CTG	Cat	Π/Ш	Ш	Ш	Ш	Ш	Ш	Ш	Ш	Ш	Ш	**#2 (Type III)**	Reference
TgCgCa1	Cougar	Ι	Π	Ш	Π	Π	Π	μ−1^a^	Ι	μ−2 a	Ι	**#66**	Reference
MAS	Human	μ−1^a^	Π	Ш	Ш	Ш	μ−1^a^	Ι	Ι	Ш	Ι	**#17**	Reference
TgCatBr5	Cat	Ι	Ш	Ш	Ш	Ш	Ι	Ι	Ι	μ−1 a	Ι	**#19**	Reference
TgCatBr64	Cat	Ι	μ-1^a^	Ш	Ш	Ш	μ−1^a^	Ι	Ш	Ш	Ι	**#111**	Reference
TgRsCr1	Toucan	μ−1^a^	Π	Ш	Ι	Ш	μ−2^a^	Ι	Ι	Ш	Ι	**#52**	Reference
TgHuZSE	Human	μ−1^a^	Π	Ш	Ш	Π	Π	Ш	Π	Π	Ι	**#9 (Chinese 1)**	This study
TgHuZS2	Human	Ι	Ι	Ι	Ι	Ι	Ι	Ι	Ι	Ι	Ι	**#10 (Type I)**	This study
TgHuAh1	Human	Π/Ш	Π	Π	Π	Π	Π	Π	Π	Π	Π	**#1 (Type II)**	This study
TgHuAh2	Human	μ−2^a^	Π	Π	Π	Π	Π	Π	Π	Π	Π	**#204**	This study
TgCtwh9, 10, 11, 12, 14, 19, TgCtxz2, 4, 6, TgCtsd1, 2, 3, 4, 5 (n = 14)	Cat	μ−1^a^	Π	Ш	Ш	Π	Π	Ш	Π	Π	Ι	**#9 (Chinese 1)**	This study
TgCtxz1	Cat	Ι	Ι	Ι	Ι	Ι	Ι	Ι	Ι	Ι	Ι	**#10 (Type I)**	This study
TgCtxz3,5,7,8 (n = 4)	Cat	Π/Ш	Π	Ι	Π	Π	Π	Π	Ι	Π	Π	**#205**	This study

a μ−1 and μ−2 represent unique RFLP genotypes, respectively.

ToxoDB#1 (type II) and ToxoDB#204 were identified from two patients with HCC in Anhui province of central China, respectively. The genotype ToxoDB#204 showed a unique pattern at the SAG1 locus and the remaining 9 loci types were identical to type II, suggesting that they are phylogenetically related. The clonal type I was also observed in humans and cats from Guangdong and Jiangsu provinces of south and east China. No clonal type III isolates were observed.

We also found four strains (TgCtxz3, 5, 7, 8) that showed a mixture of clonal types I and II patterns at some loci, while only the clonal type I pattern could be noted at the loci SAG3 and L358, clonal type II alleles at SAG2, BTUB, GRA6, c22-8, c29-2, PK1, and Apico, as well as a mixture of types II and III patterns at the SAG1 locus. These four isolates, which had not been described before, were designated ToxoDB#205. The genotypes of both ToxoDB#204 and ToxoDB#205 were found for the first time among the isolates analyzed to date in China (Table 2).

**Figure 1:**
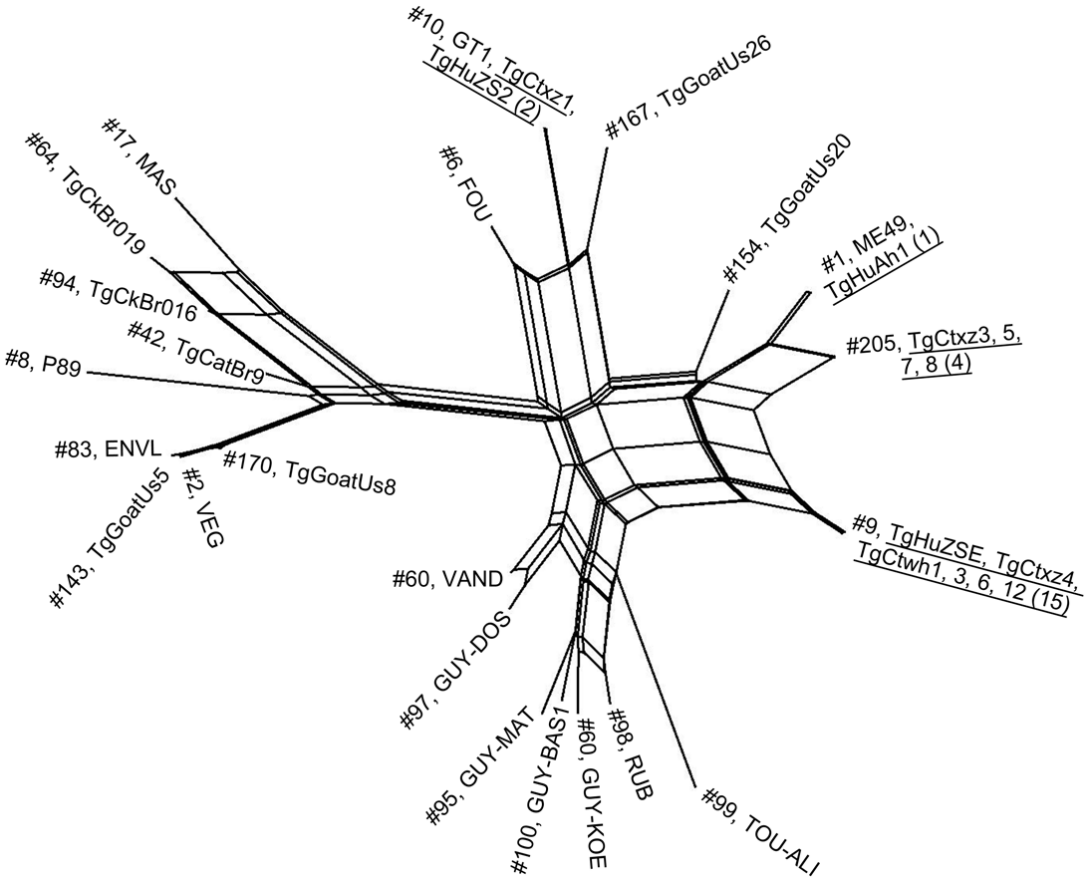
Phylogenetic network analysis of *Toxoplasma gondii* isolates from humans and animals in China. From the 23 samples, 5 genotypes were identified. Genotype number (ToxoDB PCR-RFLP #) and the representative strains are listed for each taxonomic branch. The representative strains from each genotype were underlined found in this study. The numbers in parentheses indicate the number of isolates from this study belonging to that genotype. The phylogenetic network analysis with EF1, HP2, UPRT1, UPRT7, GRA6 and GRA7 shows a perfect match with PCR-RFLP allele types at 10 multilocus markers used in the present study.

The phylogenetic network analysis of representative isolates of each genotype in this study and 21 references are summarized in Figure 1. DNA sequences of representative strains with Chinese 1 lineage are identical, and there is no variation based on the sequence data generated. The phylogeny-based concatenated sequencing also shows that four isolates (TgCtxz3, 5, 7, 8) with type ToxoDB#205 were closely related to strain ME49 (genotype II, ToxoDB#1).

### Mouse Virulence of the Chinese *T. gondii* Isolates

The mortality and the duration of survival post infection in mice inoculated with the representative strains of each genotype were shown in Table 3. Genotype ToxoDB#9 (Chinese 1) included 15 isolates from four provinces that are over 400 km apart. The mortality of mice varied from 10% to 100% by peritoneal inoculation with 1 000 tachyzoites of genotype Chinese 1. Mice inoculated with TgCtwh6, TgCtsd2 and TgCtsd3 tachyzoites, however, developed a dominated form of disease and parasites were visualized in the peritoneal lavage between 10–14 days post infection. Tissue cysts could be found in the brains of all the survivors challenged with the three isolates on day 45 post infection. Comparatively, 100 percent of mice were killed within 5–8 days after peritoneal inoculation by genotype Chinese 1 isolates (TgCtwh12 and TgCtwh14), and the similar time of death as ToxoDB#205 was noted in TgCtxz5 and TgCtxz8 isolates. The present observation, together with the previous report [28], suggests that the parasite strains with genotype Chinese 1 have varied phenotypes of virulence to mice.

**Table 3:** Summary of mouse virulence of representative strains with differents genotypes from China.

Isolates ID	Location	% Mortality in mice(No. died/No. infected)	Days of survival post infection	Genotypes	Comments
RH		100 (10/10)	5–7	#10, Type I	
PRU		0 (0/10)	Survived	#3, Type II	
TgCtwh6	Wuhan Hubei	0 (0/10)	Survived	#9, Chinese 1	this study
TgCtwh12	Wuhan Hubei	100 (10/10)	6–8	#9, Chinese 1	This study
TgCtwh14	Wuhan Hubei	100 (10/10)	5–6	#9, Chinese 1	This study
TgCtsd2	Jinan Shandong	10 (1/10)	Survived	#9, Chinese 1	This study
TgCtsd3	Jinan Shandong	0 (0/10)	Survived	#9, Chinese 1	This study
TgCtxz1	Xuzhou Jiangsu	100 (10/10)	3–4	#10, Type I	This study
TgCtxz5	Xuzhou Jiangsu	100 (10/10)	5–6	#205	This study
TgCtxz8	Xuzhou Jiangsu	100 (10/10)	5–7	#205	This study

### Kinetics of Dissemination Following Oral Infection with TgCtwh6 Isolate with Genotype Chinese 1

**Figure 2:**
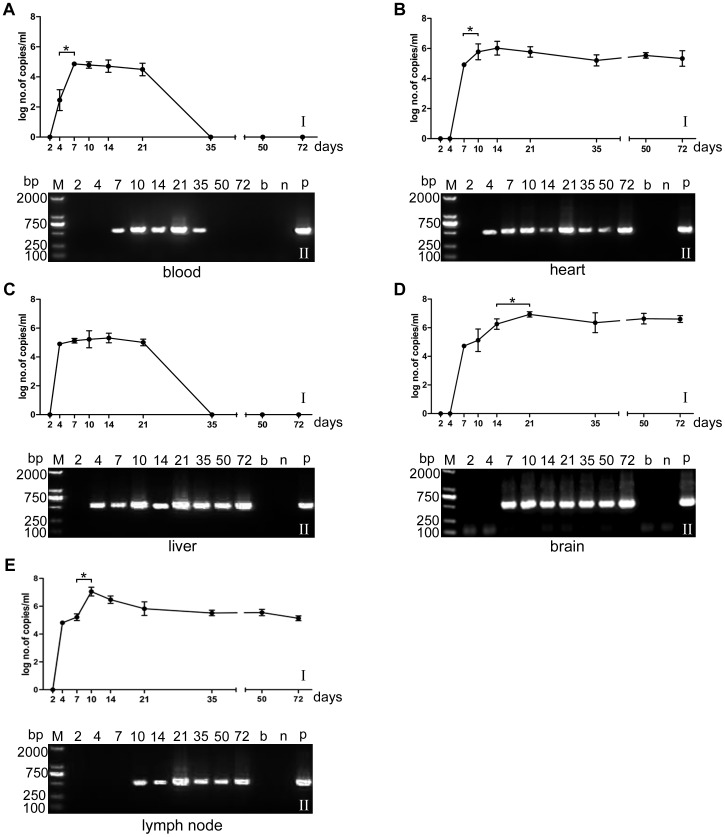
The kinetics of infection in mice with TgCtwh6 isolates. Kinetics of infection in blood and organs after oral infection with 50 cysts of TgCtwh6 isolate (genotype Chinese 1) in mice. I: the number of DNA copies in blood and tissues of the TgCtwh6 isolate post infection at various intervals by qPCR. Each point represents the mean value of the *T. gondii* DNA copies for five mice ± SD. II: detection of PCR products of *T. gondii* 529 bp fragments extracted from brain or ascitic fluid in the recipient mice. Abbreviations: **A**; blood, **B**; heart, **C**; liver, **D**; brain, and **E**; lymph node. b, n, and p represent blank, negative and positive control. *P<0.05.

As shown in Figure 2, when mice were infected with 50 cysts of the TgCtwh6 isolate, Toxoplasma DNA became detectable from day 4 in the blood, liver and lymph node tested by qPCR. Parasites were also demonstrated in the heart at day 4 by the second mouse passage. In brain tissues, DNA copies were at a low level at day 7 (5.52×10^4^ copies/ml) and significantly increased from day 14 to day 21 (*P*<0.05), then remained at a stable level thereafter. The brain cysts were firstly visualized from day 21, gradually increased in quantity and size with the time of infection and then kept in a stable level. Similar results were obtained in the heart tissues. At day 7, the number of parasites in lymph nodes were massively increased (*P*<0.05) and reached the peak at day 10 (1.33×10^7^ copies/ml), then gradually decreased to day 72 when the experiment ended. Parasitemia could be demonstrated by qPCR from day 4 and became negative at day 35, whereas cysts still could be noted in the recipient mice of second passage with blood. In the livers, parasite burden progressively increased from day 4 to day 10 and then gradually decreased from day 14 and became negative at days 35. Parasites, however, were still notable in the liver by mice passage at days 35, 50, and 72 (Figure 2).

## Discussion

Recent studies demonstrated that the global pattern of *T. gondii* population structure is more complex than previously suggested [10]. In this study, genotyping of *T. gondii* strains from humans and animals revealed the limited diversity within and between *T. gondii* populations and the dominance of Chinese 1 (ToxoDB#9) lineage in China. This genotype diverges from the three predominant clonal archetypes in North America and Europe, commonly termed as type I, II, and III by PCR-RFLP and multi-locus enzyme electrophoresis.

**Figure 3:**
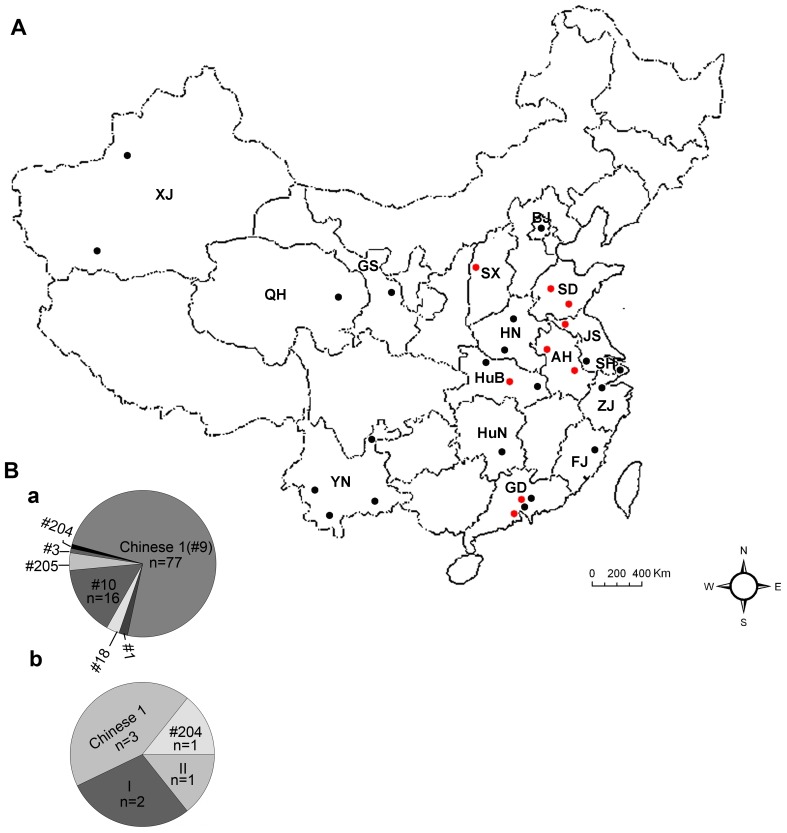
The genotypes and geographic regions of *T. gondii* Chinese isolates. Panel A: a map of China to show the cumulative data of geographic distribution where the Chinese isolates of *T. gondii* used for genotyping were collected. The provinces and cities where *T. gondii* isolates were obtained and indicated by red dots and black dots, respectively. Red dots represent the locations (counties) where the isolates were collected in this work. Black dots indicate the locations (counties) where the isolates were collected in the early studies. SX: Shanxi province; SD: Shandong province; HN: Henan province; AH: Anhui province; JS: Jiangsu province; HuB: Hubei province; HuN: Hunan province; GD: Guangdong province; ZJ: Zhejiang province; FJ: Fujian province; YN: Yunnan province; QH: Qinghai province; GS: Gansu province; XJ: Xinjiang province; BJ: Beijing city; SH: Shanghai city. Panel B: a: the cumulative data of genotypes of *T. gondii* Chinese isolates. b: the genotypes of *T. gondii* isolates from human patients in mainland China. This is the first report of the genotype ToxoDB#204 from humans.

The magnitude of the diversity in the isolates sampled in this study was compared with the overall diversity in the composite dataset from animals and humans, including 104 isolates from sixteen regions in China (Figure 3). A total of seven genotypes, including type I (ToxoDB#10) and type II (ToxoDB#1), and additional 5 atypical genotypes, were identified from distinct areas, suggesting the limited genetic variability with a few dominant genotype of Chinese isolates of *T. gondii*. The frequency of genotype Chinese 1 is unexpected, this lineage was identified in 15/23 (65.22%) isolates in the present investigation. The genotype isolates had been previously collected and identified in cats, swine and human patients from China [23], [26], [28], [27]. Taken together, it indicates that the genotype Chinese 1 is dominantly prevalent in China. Combined with results previously [19], it suggests that the common genotype Chinese 1 corresponds to the characterized Haplogroup 13, which is widely distributed in China. Our research in Chinese 1 (ToxoDB#9) at the four introns (EF1, HP2, UPRT1 and UPRT7) is similar to that reported by other investigators based on the phylogenetic network analysis of the sequence data [28]. Here we found that the strain of TgCtwh6, and TgCtwh8 as well, being capable of forming cysts in the brains, shared the same patterns as the other virulent members of type Chinese 1 at all 10 loci, including SAG3, BTUB, and GRA6 through triplicate genotyping. It might be a misreading due to incomplete digestion of the PCR-generated fragments in the previous experiment [26].

Genotyping of Chinese *T. gondii* isolates from immunocompetent individuals is challenging due to the chronic nature of infection characterized by tissue cysts and an absence of circulating tachyzoites. This parasite, however, can enter blood stream and become detectable in active infection. Studies of human toxoplasmosis in Europe show the dominance of type II strains and type I infection is rare [14], [29]. However, surveys on samples of this parasite from patients in China have revealed the novel genotypes and predominant type Chinese 1. The genotype determined in these clinical samples has been previously identified from swine and cats in China [23], [24], [26], suggesting the importance of these animals serving as the reservoirs for human infection. Combining the previous findings together with the present investigations, genotypes in human cases have been identified as Chinese 1 (3/7, 42.86%), ToxoDB#204 (1/7, 14.29%), type I (2/7, 28.57%), and type II (1/7, 14.29%) [27] (Figure 3). The novel genotype, ToxoDB#204, which has not been reported previously, shows a unique pattern at the SAG1 locus and the remaining 9 loci types are the same as type II, suggesting a genetic recombination among these populations in China. These results added information to Chinese *T. gondii* isolates prevailing in humans and showed that use of nine loci for genotyping is insufficient to obtain reliable structural data. The TgHuAh1 isolate was identified as clonal type II (ToxoDB#1), this is the first report of ToxoDB#1 strain from humans in China, although an isolate from sheep and birds, in which the type I Apico locus was notably different, was designated as type II (ToxoDB#3) in the previous literature [27], [30]. No clonal type III strains were identified among these isolates.

The analysis of population structure of Chinese *T. gondii* isolates revealed that a successful clonal lineage has expanded to wide regions of mainland China. Genotype Chinese 1 (ToxoDB#9) has also been found from Sri-Lanka, Vietnam, Colombia, Brazil, and the USA as well [31], [32], [33], [34]. Taken together, it indicates that genotype Chinese 1 is wide spread across a few continents and is the predominant isolate in China. Comparison of the identified genotypes of isolates between felids and swine showed obvious differences although Chinese 1 and ToxoDB#10 (type I) were found in both species of animals. Genotypes ToxoDB#205 and ToxoDB#18, however, have not been identified in swine to date [23], [24], [27]. This may suggest a host preference of this apicomplexan with genetic diversity in China although the standpoint needs to prove with more additional samples.

Among the isolates typed in this study, four are of clonal type ToxoDB#205, a newly described genotype and no RFLP Genotype Number is currently recorded in the Toxo-genotyping database. Genotype ToxoDB#205 seems to be a recombination of *T. gondii* type I and II and most related to type II, indicating that it may have been derived by sexual recombination of these two clonal types in the domestic animals in China. Sexual recombination may lead to the generation of new genotypes with an altered biological trait, such as an increased mouse virulence, as shown previously [35], [36], and could also result in new genotypes of *T. gondii* with a higher virulence to humans.

The virulence of *T. gondii* isolates usually shows difference according to their geographic region and the host. The archetypal strains of *T. gondii* show a difference of virulence in mice: type I isolates were found to be acutely virulent to mice (LD_100_ = 1 parasite), whereas types II and III were relatively avirulent and can establish latent infection (LD_100_>1000 parasites) [11], [37]. At present, little is known about the association of genetic type with virulence to mice for *T. gondii* from animals and humans in China. In this study, the virulence of representative *T. gondii* strains of each genotype was determinated by intraperitoneal inoculation with 1×10^3^ tachyzoites to mice and showed intermediately to highly virulent. Interestingly, mice infected with TgCtwh6, TgCtsd2 and TgCtsd3 isolates, that genetically share the type Chinese 1, had a relatively low virulence to mice and cysts could be visualized in abundance in the brain of mice after 3 weeks post inoculation. Similar phenomenon could also be noted in other studies on genotype Chinese 1 isolates [28]. The present result suggests that the ten molecular markers currently used for *T. gondii* genotyping may be insufficient to differentiate the strain virulence when used for genotyping of the isolates with type Chinese 1. The mouse virulence differences among isolates with the same genotype Chinese 1 should not be neglected and the genotypic configuration may not be deemed to be deservedly associated with their real determinants of virulence in *T. gondii* isolates in China. Obviously, more studies need to be carried out on such an interesting observation.

So far, few pathogenically confirmed human cases of toxoplasmosis have been presented in China although a relatively high sera prevalence (7.9%) of *T. gondii* was reported during the past decades [21], [38]. Kinetics of *T. gondii* in human infection is fundamental to understanding the dissemination patterns, pathogenesis, and parasite virulence. However, little is known regarding the biological characteristics of the strain of *T. gondii* with genotype Chinese 1 which is able to form cysts in the tissues. In the present work, parasitemia was confirmed by mouse passage and qPCR. Our results showed that the parasitemia of TgCtwh6 in mice persisted for at least 35 days post infection, which might facilitate the dissemination of the parasites to other tissues of the body and the transmission of the parasites through the placenta to a developing foetus. Previous study [11], [39], [40] has also indicated that virulent isolates could induce high parasitemia which can increase the transmission through the placenta and cause severity diseases in developing foetus. The presence or absence of parasites in blood and the duration of parasitemia are additional signs of virulence since it is detectable in acutely infected mice but not demonstrable during latent infection. The present approach provided an evidence for the tissue distribution of *T. gondii* in mice and preliminary information for further study of pathogenesis.

In conclusion, our results revealed a limited genetic diversity among isolates of *T. gondii* from different geographic areas from both animals and humans in China and a dominant genotype Chinese 1 and two novel genotypes, ToxoDB#204 and ToxoDB#205, have been described. Further studies on *T. gondii* from domestic and wild mammals, birds, and humans are necessary for a better understanding of the potential relations between the population structure diversity and its pathogenesis in Chinese *T. gondii* isolates.

## Materials and Methods

### Ethics Statement

Ethical permission was obtained from the Institutional Review Board (IRB) of the Institute of Biomedicine at Anhui Medical University (Permit Number: AMU26-080610), which records and regulates all research activities in the school. The IRB of the Anhui Medical University Institute of Biomedicine approved both animals and humans protocols. The approval from the IRB also includes the permission of using the stray cats under euthanasia and all experimental procedures carried out in strict accordance with the recommendations in the Guide for the Care and Use of Laboratory Animals of the National Institutes of Health. All of the human participants had been informed about the aim and procedures of the study and provided their written informed consent or, in case of illiterates, oral consent prior to examination. The use of oral consent had been specifically approved by the IRB. For the illiterates, the protocol of the study, the rights and the potential benefits and risks of the participants were read over to them, followed by their personal signing on the sheet after agreement: writing names of their own if they are able to, or pressing the fingerprint of right hand if they are not able to. The documents with participants’ signs were in duplicate. The IRB and the participant each have one copy.

### Serological Tests

To establish the diagnosis and rule out other disease etiologies, sera were collected and all suspected cases of toxoplasmosis were tested. One hundred and eighteen sera samples were collected from patients in Anhui and Guangdong from August 2010 to September 2011. Diagnosis of toxoplasmosis was based on clinical and laboratory tests. The laboratory methodologies included: (i) serological tests of specific anti-Toxoplasma IgG and IgM antibodies by the indirect and capture ELISA; (ii) detection of Toxoplasma circulating antigen (CAg) by the double antibody sandwich ELISA; and (iii) conventional PCR targeting at the highly conserved 529 bp repeat element in the genome of *T. gondii* [41]. The ELISA was performed using a commercial test kit (ACON, China) to determine *T. gondii* antibodies and CAg according to the manufacturer’s instructions. The reaction cut-off was calculated as the mean optical density (OD) for negative control sera plus three standard deviations. The positive and negative control sera were included in each plate and were obtained from the kit. The reading was performed using a microplate reader (Bio-Tek, USA) set at a level of absorbance of 450 nm. All samples were run in triplicate. The results were considered positive when OD_450_ index was equal or higher than the cut-off value in ELISA. Blood samples from sera positive human patients were bioassayed in mice for viable parasite isolation and extraction of DNA for molecular typing.

### Animal Samples and Bioassay in Mice

A total of 105 stray cats were collected from Hubei (31), Shandong (24) and Jiangsu (50) Provinces, respectively. All cats were anesthetized before being sacrificed.

Brain and heart samples of all cats were bioassayed in 5-week-old Swiss Webster (SW) mice (specific pathogen free, SPF), obtained from the Anhui Laboratory Animal center, for *T. gondii* examination and isolation, as previously described [42]. Briefly, 100 grams of samples of tissue (brain and heart) from each animal were homogenized in 250 ml of 0.9% (W/V) saline containing antibiotics (penicillin 1000 U/ml, streptomycin 100 µg/ml), mixed with identical volume of pepsin solution and the mixture was incubated in a shaking water bath for 1 h at 37°C. The suspension was then filtered, centrifuged, neutralized and mixed with antibiotics, and the homogenate with 1 ml from each cat was inoculated intraperitoneally into each of five mice. Inoculated mice were kept in a room with controlled photoperiod and temperature, and fed with water and commercial mice pellets. Peritoneal exudates were examined from mice for viable *T. gondii* tachyzoites as soon as obvious clinical manifestations (weight loss, ruffled fur, hunched posture and lethargy) were observed, and survivors were sacrificed under anesthesia on day 45 post inoculation and the brains squashes were examined microscopically for tissue cysts [1]. The viable tachyzoites or cysts from infected mice were cryopreserved in liquid nitrogen [43] and used for purification of *T. gondii* genomic DNA for multilocus genotyping studies. Mice were considered infected with *T. gondii* when tachyzoites or cysts were demonstrable in their tissues.

### DNA Extraction and Genotyping of *T. gondii* Isolates

The extraction and purification of *Toxoplasma gondii* DNA from ascitic fluid and/or brain tissues of infected mice were carried out by using the commercial kit QIAamp® DNA Mini kit (QIAGEN, Germany) and with proteinase *K* at a final concentration of 1 mg/ml, according to the manufacturer’s instructions. The samples were incubated at 56°C in water bath for 1–3 h and vortexed several times during incubation. Genomic DNA was eluted in 100 µl of AE buffer provided with the kit and stored at −20°C until use.

Genotyping of *T. gondii* isolates was performed using multilocus PCR-restriction fragment length polymorphism (RFLP) and 10 genetic markers, including 9 nuclear loci, SAG1, SAG2, SAG3, BTUB, GRA6, L358, PK1, c22-8, c29-2, and one apicoplast locus Apico, as previously reported [44], [45]. All *T. gondii* strains isolated from animals and humans were submitted for genotyping. Furthermore, 14 *T. gondii* DNA samples originating from an earlier collection [26] were also included and re-genotyped. These had been derived from cats from different areas in China: Anhui (two samples), Guangdong (two samples), Shanxi (two samples), and Hubei (eight samples) provinces. Reference strains of *T. gondii* were used as controls for genotyping, including type I (GT1), type II (PTG), type III (CTG) and other strains (TgCgCa1 (a.k.a. Cougar), MAS, TgCatBr5, TgCatBr64, TgRsCr1). Briefly, the target DNA sequences were amplified by PCR using PCR Master Mix (Promega, USA) for all markers. Each PCR reaction was conducted on 1.5 µl (100 ng) of each DNA extraction sample with 25 µl of PCR Master Mix, 50 pmol of each primer with the total reaction volume reaching 50 µl. The reactions were performed in a thermal cycler (Biometra, Germany) with an initial denaturation step of 94°C for 5 min, followed by 38 cycles of denaturation at 94°C, annealing temperatures appropriate for the primer, and extension at 72°C for 1 min each and a final 10 min incubation at 72°C to complete partial polymerisation. Primer annealing temperatures were derived from previous studies [26], [45]. Five microliters of each PCR amplification product were visualized on 1% agarose gels stained with ethidium bromide dye. In order to achieve higher quality result the remaining PCR products from each marker and sample were digested with restriction enzymes after purification using AxyPrep™ PCR Cleanup Kit (Axygen, USA) according to the manufacturer’s instructions. Restriction enzymes were purchased from Fermentas, Germany. Digestion with endonucleases was performed at a final volume of 20 µl, consisting of 10 µl of PCR purified products, 2 µl of the respective buffer and 1 µl of the enzyme at the correct temperature for each restriction enzyme, in accordance with the manufacturer’s guidance. The digestion products were analyzed by gel electrophoresis in 2.5 or 3% high resolution agarose at 65 V for 70 min, depending on the marker, stained with ethidium bromide and recorded using digital gel documentation system (Bio-Rad, USA).

Each PCR-RFLP analysis included a positive and negative control alongside samples to be analyzed. A low range DNA ladder (25–700 bp) (Fermentas, Germany) was used as a size standard. The primers and enzymes used were stated previously [44], [45].

### DNA Polymorphism Analysis

PCR products for representative strains from each genotype identified in this study were sequenced at four introns (EF1, HP2, UPRT1 and UPRT7) as described previously [46], combined with two dense granule proteins (GRA6 and GRA7). Three cat isolates (TgCatBj1, TgCatBj3 and TgCatBj7) previously reported from Beijing were included in this study [28] to compare the DNA sequence data with the present study at loci EF1, HP2, UPRT1 and UPRT7. The target sequences were amplified by PCR. Amplified PCR products were purified and sequenced from one end using sequencing primers. Sequences were aligned with each other and with previously published sequences [46], [47], using Bioedit software (available free-of-charge at http://www.mbio.ncsu.edu/BioEdit/bioedit.html). NeighborNet phylogenetic network of representative isolates with different genotypes and reference strains were inferred using the software SplitsTree v4.4 [48]. GenBank database accession numbers of reference strains used for phylogenetic network analysis are shown in Table S1.

### Mouse Virulence Determination of *T. gondii* Isolates

To determine the association between multilocus genotypes and virulence phenotypes in mice, ten SW mice (SPF) were intraperitoneally inoculated with 1000 tachyzoites of representative Chinese *T. gondii* strain from each genotype. Parasites were harvested from the peritoneal cavities of mice infected 3–4 days earlier by aspiration except TgCtwh6, TgCtsd2 and TgCtsd3, at 10–14 days post infection. For the purification of tachyzoites, the solution containing *T. gondii* and host cells were centrifuged at 45×g for 5 min at 4°C. The supernatant was centrifuged at 1350×g for 10 min at 4°C, and then suspended in phosphate buffered saline (PBS) at a concentration of approximately 1×10^4^ parasites/ml [49]. The mice were observed for 45 days as in mice bioassay. The virulence was defined based on the response variables of presence or absence of peritoneal fluid, cumulative mortality, and the number of days of survival of animals infected [35]. Cumulative mortality was defined as the number of deaths/number of animals infected.

### Assessment of Kinetics of Dissemination Following Oral Infection with TgCtwh6 Isolate

Kinetics of the TgCtwh6 isolate (Chinese 1 lineage) dissemination in blood and tissues of SW mice were also investigated by fluorescence quantitative real time PCR (qPCR) and subinoculation into fresh mice. Mice were orally inoculated with 50 cysts, which had been isolated from the entire brain of infected mice by density gradient centrifugation over Fycoll-paque plus as previously described [50]. Peripheral blood, lymph nodes, heart, liver and brain of mice were collected on days 2, 4, 7, 10, 14, 21, 35, 50, and 72 post infection and 5 mice were sacrificed under anesthesia at each time of collection. The blood was collected from the retroorbital sinus with sodium citrate. Each tissue was collected from the same organ at the same day post infection and gently wiped on sterile gauze, weighed and then homogenized with antibiotics [39]. The homogenates were independently filtered through one layer of gauze and were separately intraperitoneally inoculated into fresh mice. The parasites were detected in recipient mice via: (i) microscopical examination of impression smears of ascitic fluid that died during acute period for tachyzoites; (ii) brain tissue squash examination for cysts that survive 45 days post infection; and (iii) DNA extraction from brain or ascitic fluid and detected by PCR with the same volume as described above. PCR tests were performed by using the primers as previously described [41] which amplify a repeated and specific 529 bp gene (GenBank accession numbers AF 487550 and AF 146527), repeated 200–300 times in *T. gondii* genome. The parasite burdens in blood and tissues during acute and chronic phase of infection were assessed in 96-wells plates using an ABI 7300 Sequence Detection System (Applied Biosystems, USA) and *T. gondii* PCR fluorescence diagnostic kit (DAAN Gene, China). The forward primer (Toxo-F), reverse primer (Toxo-R), and TaqMan probe for qPCR amplification were provided in the kit. Routine vigilances were seriously used to avoid the risk of contamination by exogenous DNA or PCR product carryover [51]. In brief, the DNA extraction was performed from 200 µl peripheral blood and 30 mg tissues of liver, heart, brain and lymph nodes, respectively. DNA was eluted in 100 µl AE buffer and subsequently 5 µl of eluted extract was used for qPCR test. The cycling parameters were: after denaturation of DNA for 2 min at 93°C, amplification was done in 40 cycles (10 cycles including 93°C for 45 s and 55°C for 1 min, plus 30 cycles of 93°C for 30 s and 55°C for 45 s). The standard curve for *T. gondii* DNA quantification was prepared using 10-fold serial dilutions of known copy numbers (10^7^ copies/ml –10^4^ copies/ml) from the kit. Plates included positive and negative controls in order to achieve higher quality control. The cycle threshold values (Ct) were set up automatically. The regression coefficient and the slope of the standard curves were calculated in order to estimate the efficiency of the assay. *T. gondii* quantities of the samples were calculated according to the standard curve, and results were expressed as copy numbers per ml of sample. The parasites were considered present when tachyzoites, cysts and qPCR-generated products were positive in recipient mice.

### Statistical Analysis

Clinical and laboratory diagnoses were used to establish human patient status. Copy numbers of parasite were compared using one-way ANOVA followed by Student-Neuman-Keuls (SNK) tested at levels of significance of *P*<0.01 and 0.05. The genotypes were compared, identified and matched to those listed in ToxoDB genotyping database at http://toxodb.org/toxo/. New genotype are assigned to new ToxoDB genotype # and deposited to the database for future reference.

## Supporting Information

Table S1 **Summary of GenBank accession numbers used for phylogenetic network analysis of reference** ***T. gondii*** **strains used in this study.** Abbreviations: ND; no accession number from ToxoDB.
(DOC)
