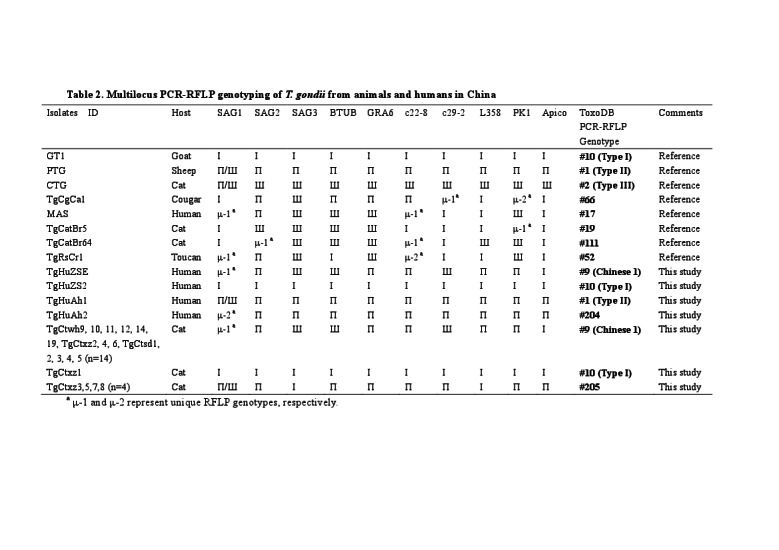# Correction: Genotypes and Mouse Virulence of *Toxoplasma gondii* Isolates from Animals and Humans in China

**DOI:** 10.1371/annotation/2cc13197-e6fc-46eb-88d6-853be10b72c5

**Published:** 2013-05-30

**Authors:** Lin Wang, He Chen, Daohua Liu, Xingxing Huo, Jiangmei Gao, Xiaorong Song, Xiucai Xu, Kaiquan Huang, Wenqi Liu, Yong Wang, Fangli Lu, Zhao-Rong Lun, Qingli Luo, Xuelong Wang, Jilong Shen

There were typographical errors in Table 2.

A correct version is available here: 

**Figure:**