# Supplementary material for: Genotypes and Mouse Virulence of Toxoplasma gondii Isolates from Animals and Humans in China
Source: PLoS One. 2013 Jan 7;8(1):e53483. doi: 10.1371/journal.pone.0053483 (PMC3538538; doi:10.1371/journal.pone.0053483)
Supplement: Table S1 — Summary of GenBank accession numbers used for phylogenetic network analysis of reference T. gondii strains used in this study. Abbreviations: ND; no accession number from ToxoDB. (DOC) [file pone.0053483.s001.doc]

**Table S1.**

| Isolates | GenBank accession numbers | | | | | |
| --- | --- | --- | --- | --- | --- | --- |
|  | EF1 | HP2 | UPRT1 | UPRT7 | GRA6 | GRA7 |
| TgGoatUs20 | HQ852148 | HQ852163 | HQ852168 | HQ852173 | HQ852153 | HQ852158 |
| TgGoatUs8 | HQ852147 | HQ852162 | HQ852167 | HQ852172 | HQ852152 | HQ852157 |
| TgGoatUs5 | HQ852146 | HQ852161 | HQ852166 | HQ852171 | HQ852151 | HQ852156 |
| TgGoatUs26 | HQ852149 | HQ852164 | HQ852169 | HQ852174 | HQ852154 | HQ852159 |
| TgCkBr016 | JQ679569 | JQ679667 | JQ679827 | JQ679947 | EF512263 | EU157180 |
| TgCkBr019 | JQ679597 | JQ679670 | JQ679828 | JQ679936 | EF512255 | EU157167 |
| RUB | JQ679611 | JQ679732 | JQ679869 | JQ680027 | AF239290 | DQ459450 |
| ENVL | JQ679541 | JQ679645 | JQ679775 | JQ679912 | EF512222 | EU157149 |
| TOU-ALI | JQ679620 | JQ679753 | JQ679830 | JQ680029 | EF512242 | EU157181 |
| GUY-KOE | JQ679535 | JQ679731 | JQ679864 | JQ680012 | EF512233 | EU157163 |
| GUY-DOS | JQ679538 | JQ679730 | JQ679761 | JQ679999 | EF512232 | DQ459451 |
| GUY-MAT | JQ679617 | JQ679722 | JQ679862 | JQ680000 | EF512231 | EU157162 |
| GUY-BAS1 | JQ679613 | JQ679729 | JQ679865 | JQ679956 | EF512225 | EU157157 |
| GT1 | JQ679537 | JQ679631 | JQ679816 | JQ679975 | JX044183 | JX045574 |
| P89 | JQ679550 | JQ679638 | JQ679874 | JQ679982 | EF512240 | JX045616 |
| VEG | JQ679551 | JQ679640 | JQ679774 | JQ679906 | JX044209 | JX045618 |
| TgCatBr9 | JQ679531 | JQ679649 | JQ679845 | JQ679954 | JX044204 | JX045628 |
| FOU | JQ679539 | JQ679727 | JQ679842 | JQ679971 | AF239288 | JX045576 |
| VAND | JQ679520 | JQ679641 | JQ679768 | JQ680006 | JX044199 | JX045600 |
| ME49 | ND | ND | ND | ND | AF239285 | JX045583 |
| MAS | JQ679607 | JQ679757 | JQ679817 | AY143153 | AF239289 | DQ459456 |
